# Primary mycetoma of the calcaneum: Case report on an unusual hazard of barefoot walking

**DOI:** 10.1016/j.ijscr.2023.108418

**Published:** 2023-06-17

**Authors:** Muhammad Umar Nasir, Muhammad Umer Mukhtar, Zoha Nasir, Qasim Mehmood, Muhammad Salar Raza, Muhammad Nasir Ali

**Affiliations:** a King Edward Medical University, Pakistan; b Quaid-e-Azam Medical College, Pakistan; c Sheikh Zayed Medical Complex, Pakistan

**Keywords:** Primary mycetoma, Chronic heel pain, Osteomyelitis, Barefoot walking, Calcaneum

## Abstract

**Introduction:**

Mycetoma is a rare tropical fungal infection characterized by a clinical triad of subcutaneous swelling, multiple discharging sinuses, and a purulent discharge containing granules. If left untreated, the disease can progress from cutaneous to intraosseous and can cause osteomyelitis. In very rare instances labeled “primary mycetoma”, the fungus is insidiously inoculated directly into the bone and causes osteomyelitis without any preceding cutaneous involvement. This can make the diagnosis very difficult.

**Presentation of case:**

A twelve-year-old girl with a history of walking barefoot, presented with pain and inability to bear weight on her left foot. There was no overlying cutaneous involvement. X-ray showed an osteolytic lesion in the calcaneum. After the failure of antibiotic treatment, the diseased bone was excised. Black granules were discovered inside the lesion and their histopathology confirmed a diagnosis of primary eumycetoma. After some time, the disease relapsed, necessitating another debridement. This occurred many times with worsened severity in each successive episode. Because of worsening disease and failure of both antifungal and surgical treatment, foot amputation was done.

**Discussion:**

Primary mycetoma is an insidious fungal infection that causes osteomyelitis without any cutaneous findings. Timely diagnosis and treatment provide the best chance of preventing an amputation.

**Conclusion:**

A high index of suspicion must be maintained for patients presenting with symptoms of osteomyelitis without any skin involvement so that timely diagnosis and treatment can prevent the progression of the disease and the need for amputation.

## Highlights

• Mycetoma is a fungal infection characterized by subcutaneous swelling, discharging sinuses, and purulent discharge.
• Rarely, the pathogen may inoculate directly into the bone resulting in a very difficult diagnosis.
• Untreated disease can progress to refractory osteomyelitis that may necessitate amputation
• Timely diagnosis and treatment provide the best chance of preventing an amputation.

## 1 Introduction

Mycetoma is a rare chronic infectious disease of the subcutaneous tissue. It affects the lower extremities, especially the foot [1]. The culprit agent can either be a fungus (Eumycetoma) or a bacterium (Actinomycetoma) [2]. This disease occurs mostly in the tropics and subtropics [3] with the highest incidence in young people. After inoculation (e.g., by a thorn), the initial lesion usually involves the skin and the subcutaneous tissue and presents as a triad of painless subcutaneous swelling, multiple discharging sinuses, and purulent discharge containing granules. Neglected disease can spread and involve deeper tissues like tendons, muscles, or bones and can result in severe disability [1,4].

In some rare instances, direct inoculation of the infectious agent into the bone occurs and results in osteomyelitis without any preceding superficial involvement. This unique presentation of the disease is known as primary mycetoma and has been seen less than ten times in literature [5,6] with most of the reported cases dating back about half a century. The unexpected and insidious involvement of deep tissues without any cutaneous manifestations makes primary mycetoma difficult to diagnose and prone to neglect. Progression of disease due to neglect can necessitate amputations. Here, we present a novel case of primary mycetoma of the calcaneus in a young girl who presented without any history of inoculation or superficial involvement. The goal of our paper is not only to report a rare and unique case but also to bring wider recognition to primary mycetoma so that timely intervention can prevent amputations in victims of this disease.

This case has been reported in accordance with the SCARE guidelines [13].

## 2 Presentation of case

A twelve-year-old female presented on April 19, 2021, with the complaint of pain, limp and inability to bear weight on the left foot for the past 3 months. Patient belonged to a rural area and was habitual of walking barefoot. On exam, patient had deep tenderness at the inferolateral aspect of the heel.

**Fig. 1:**
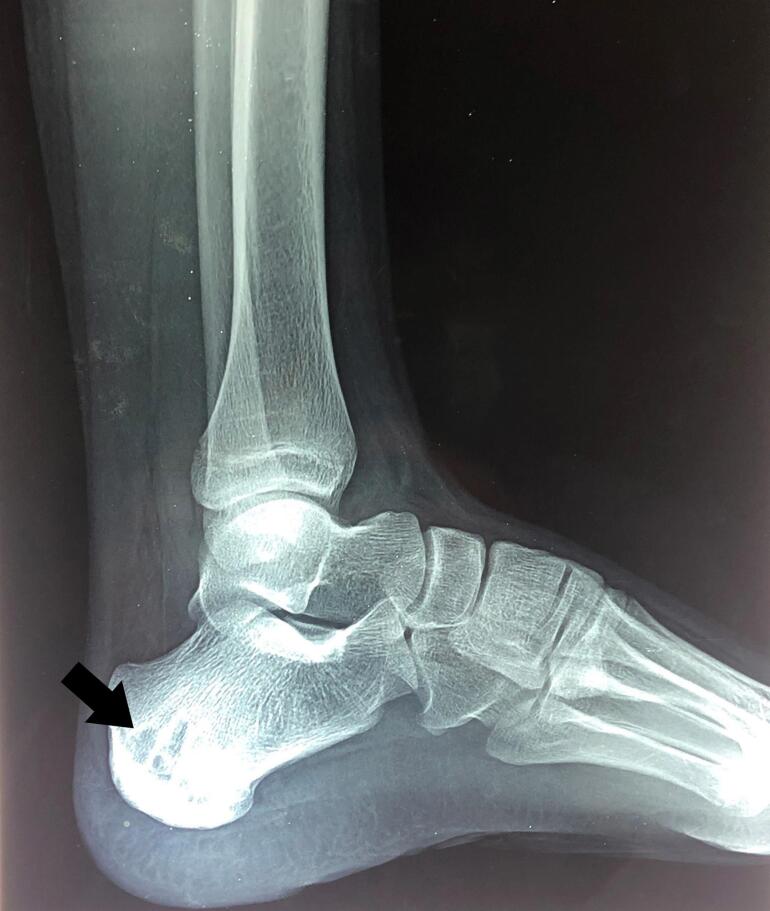
Lateral radiograph of left foot showing an osteolytic lesion in the calcaneum (arrow).

**Fig. 2:**
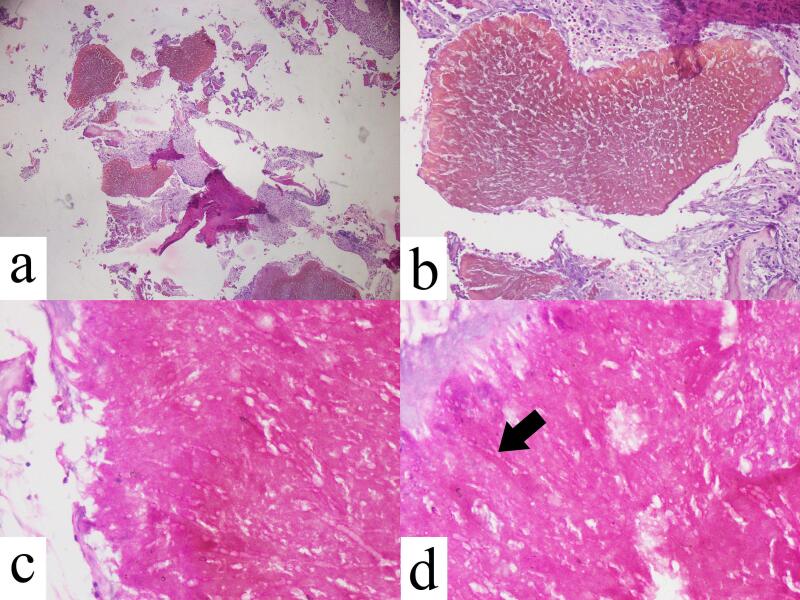
Histopathological findings at various magnification levels. Septate branching hyphae consistent with eumycetoma seen (arrow).

Given the patients' age, insignificant history and lack of any local physical findings except tenderness, differential diagnoses of Sever disease, chronic osteomyelitis and simple bone cyst were made. Her baseline investigations were within the normal range however, the lateral radiograph of the foot revealed an osteolytic lesion in the calcaneus (Fig. 1) narrowing the diagnosis down to chronic osteomyelitis and simple bone cyst. Perceiving the disease as benign, the patient was prescribed broad spectrum antibiotics, NSAIDs and silicon heel pad for her condition and after a follow-up period, she presented without any improvement. Due to the progressive and refractory nature of the disease, bone curettage and debridement was performed on September 3, 2021 with patient consent by an orthopedic surgeon with experience of 20 years. Preoperative MRI was not done due to unaffordability to the patient. Intraoperatively, there was an osteolytic lesion involving approximately one-third of the calcaneum containing multiple black granules and a decent amount of dead bone. Excisional biopsy was done. Histopathology revealed numerous pigmented fungal hyphae showing branching at right angles (Fig. 2). Based on the characteristic black granules and the microscopic features of the specimen, a diagnosis of eumycetoma was made after which antibiotics were discontinued and patient was prescribed oral antifungal therapy (itraconazole and ketoconazole, both 400 mg/day) according to culture and sensitivity results.

**Fig. 3:**
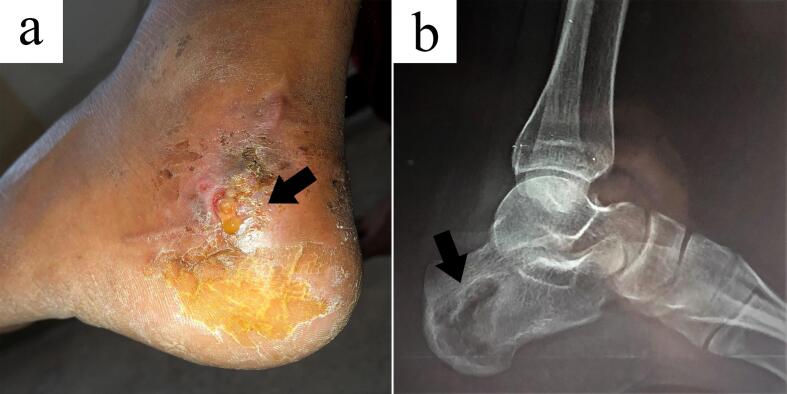
Figures showing postoperative recurrence of the disease. (A) Draining sinus seen on the posteroinferolateral aspect of the left ankle (Arrow). (B) Enlarged osteolytic lesion seen in lateral radiograph of the left foot in the same region (B).

One month later, the patient presented again with subcutaneous swelling and multiple sinuses with granular discharge at the posteroinferolateral aspect of the ankle, close to the insertion of the tendocalcaneus (Fig. 3A). X-ray revealed an increase in the size of the osteolytic lesion as compared to that before surgery, presumably due to both prior curettage and reactivation of the disease (Fig. 3B). Patient had absence of common risk factors of immunodeficiency such as HIV, diabetes and malnutrition, ruling our immunocompromise as the factor leading to the recurrence of disease.

After 2 more months of oral antifungal therapy and worsening disease progression, surgical debridement along with bone curettage was repeated and the patient was continued on the same antifungal regimen. However, the patients' condition worsened yet again leading to another debridement a month later.

**Fig. 4:**
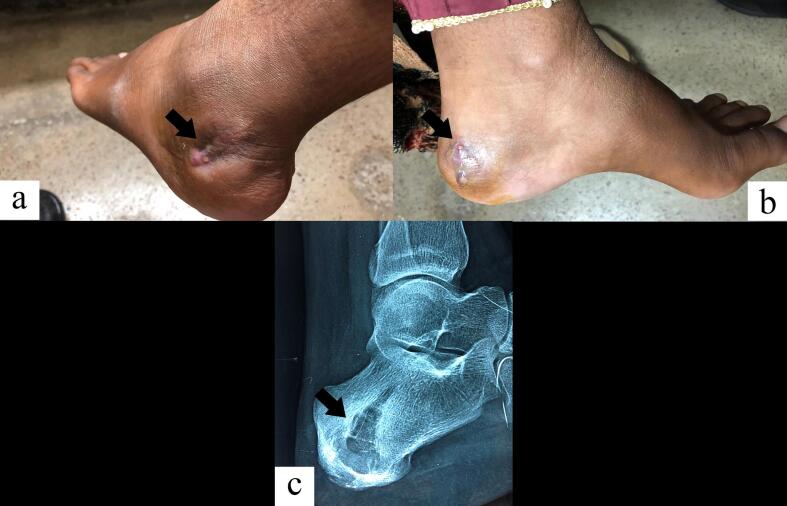
Draining sinuses seen on either side of the posteroinferolateral aspect of the left ankle (A, B). An enlarged osteolytic lesion with a cortical break seen in lateral radiograph of the left foot in the same region (C).

The most recent follow-up of the patient was on February 10, 2022 (Fig. 4). The patient had complaint of persistent pain and inability to bear weight on the left ankle. On examination, discharging sinuses and subcutaneous swellings were now present on both sides of the foot (Fig. 4A, B). X-ray revealed an enlarged osteolytic lesion extending almost across the entire calcaneum with a cortical break on the extra-articular side but no periosteal reaction or subtalar or mid-tarsal joint involvement (Fig. 4C). Owing to the failure of repeated surgery, prolonged antifungal therapy and progressive nature of the disease, the patient was given an option of total calcanectomy or Syme's amputation. The patient, however, did not give consent for any of the above procedures and failed to follow up.

## 3 Discussion

Mycetoma is a rare chronic infection with the number of reported cases being as low as 127 cases per year. It has been designated as one of the seventeen neglected tropical diseases by WHO [7]. Mycetoma is divided into two different types based on the pathogen i.e., actinomycetoma (bacterial pathogens) and eumycetoma (fungal pathogens). This classification is important as management depends on the pathogen type involved. The common organisms responsible for Actinomycetoma include *Nocardia* species (produce white to yellow granules) and *Actinomadura pelletieri* (produces red to pink granules) and common causative microbes of eumycetoma include *Madurella mycetomatis* (produces black granules), *Neotestudina rosatii* (produces yellow granules), *Fusarium* species (produce white to pale yellow granules) [2].

Actinomycetoma is distinguished from eumycetoma by its clinical features of frequent extra pedal involvement, more aggressive clinical course, multiple fistulae, earlier bone involvement with smaller cavities, more frequent lymphatic spread, and better response to chemotherapeutic treatment [8].

The causative fungi and bacteria that cause mycetoma are soil inhabitants and are inoculated into the skin through minor trauma or insignificant injury on any part of the body [9]. Mycetoma manifests as a clinical triad of painless subcutaneous swelling, multiple discharging sinuses, and purulent discharge containing granules. If the disease is not attended to, it can spread into the deeper tissues i.e. tendons, fasciae, muscles, and bones, and can lead to severe debilitation and disability [2,4]. In our case the patient presented with primary involvement of the bone without any preceding superficial lesion, a presentation consistent with a primary mycetoma.

This disease is much more common in the tropics and subtropics, with the highest concentration of the disease in the “mycetoma belt” region that compromises the latitude regions between 15 degrees South and 30 degrees North and includes Venezuela, Chad, Ethiopia, India, Mexico, Somalia, Senegal, Yemen, Thailand, and Sudan [8]. Although the disease is commonly encountered in Pakistan, it is uncommonly reported [3].

Mycetoma like many other neglected tropical diseases occurs mostly in people belonging to rural areas and with low socioeconomic status. It is uncommon in children (that compromise only 3.0–4.5 % of all endemic cases) [8] and is much more common in the age group of twenty to forty years [5]. Mycetoma has a male to female preponderance of 3:1 [2,4,10].

Although a provisional diagnosis of mycetoma can be made based on the classical clinical presentation, the diagnosis of primary mycetoma represents a challenge because the classical clinical presentation is absent. A high index of suspicion is required on the part of the investigator to arrive at the diagnosis. One of the goals of our paper is to bring wider recognition to the unique presentation of primary mycetoma so that it can be adequately diagnosed. In all cases, the diagnosis must be supplemented with studies like X-ray, ultrasound, MRI, fine-needle aspiration cytology, and histopathology. Radiography shows lytic lesions that may be small and numerous (actinomycetoma) or large and few (eumycetoma). A non-specific presentation of osteomyelitis without any discernable superficial lesions, as encountered in our case, must raise suspicion for primary mycetoma [10].

Treatment for actinomycetoma and eumycetoma can be both medical and/or surgical. A combination of trimethoprim-sulfamethoxazole (7.5–40 mg/kg divided in two doses daily) and amikacin (15 mg/kg daily) continued for weeks or months is the therapy of choice for actinomycetoma and can be supplemented with surgical excision and debridement for a better response. For eumycetoma, itraconazole (300 mg daily) and ketoconazole (400 mg daily) can be used, with voriconazole and posaconazole used in refractory cases. Antibiotic susceptibility testing is recommended but it does not always result in a corresponding response in vivo. Antifungal therapy must be supplemented with surgical excision and debridement for eumycetoma. Amputation may be needed in severe cases. Patients must be regularly followed due to the high rate of recurrence associated with surgery [11].

Late presentation or neglected disease, especially in cases of primary mycetoma, as in our case, can lead to extensive tissue destruction leading to impaired mobility and amputations. This can be severely debilitating, not only personally but also socially and economically for the young patients of this disease [12]. Thus, timely diagnosis and treatment of mycetoma are very important to prevent such complications.

## 4 Conclusion

Mycetoma primarily affects the foot and presents with a triad of painless subcutaneous swelling, discharging sinuses, and purulent discharge containing granules. Uniquely, primary mycetoma occurs due to direct inoculation into the bone and presents without the classical triad. A high index of suspicion must be maintained in patients presenting with symptoms of osteomyelitis without any skin involvement so that timely diagnosis and treatment can prevent disability and amputation.

## Consent

Written informed consent was obtained from the patient's parents for publication and any accompanying images. A copy of the written consent is available for review by the Editor-in-Chief of this journal on request.

## Ethical approval

Ethical approval for this study was provided by the Head of Department of Orthopedic Surgery, on 6 May 2023. (The institute does not grant specific protocol numbers to ethical approvals granted to case reports.)

## Funding

This study had no funding sources.

## Author contribution

**Muhammad Umar Nasir**: Conceptualization, Data curation, Writing- Original draft preparation, Writing - Review & Editing **Muhammad Umer Mukhtar**: Conceptualization, Software, Writing- Original draft preparation, Writing - Review & Editing, Visualization. **Zoha Nasir**: Writing- Original draft preparation. **Qasim Mehmood**: Project administration, Writing - Review & Editing. **Muhammad Salar Raza**: Methodology, Investigation. **Muhammad Nasir Ali**: Supervision, Project Administration, Investigation, Resources.

## Guarantor

Muhammad Umar Nasir.

## Research registration number

1. Name of the registry: UMIN-CTR.
2. Unique identifying number or registration ID: UMIN000047784.
3. Hyperlink to specific registration: https://center6.umin.ac.jp/cgi-bin/ctr_e/ctr_view_reg.cgi?recptno=R000058214.

## Declaration of competing interest

The authors have no conflict of interest to declare.
